# 

**Published:** 1891-09-12

**Authors:** 


### THE STUDENT OF MEDICINE.

[All communications for this Supplement should be addressed to THE EDITOR of THE HOSPITAL, at 140, Strand, with the words, "Students' Column," written in the left hand top corner of the envelope.]

### MEDICAL SCHOOLS AND DEGREE GRANTING CORPORATIONS.

WE give below a list of those Universities and Medical and Surgical Corporations which confer degrees and diplomas in medicine and surgery. To this is added a list of those hospitals and teaching institutions at which medical education may be obtained. It will be noted that some institutions are both educating and degree-conferring bodies. Persons desiring information about any particular University, Royal College, or Hospital Medical School, should write to the Dean of the Medical Faculty of that institution for a copy of its rules and regulations. All the Universities, Colleges, and Hospitals named are anxious to secure new students, and they are therefore always very willing to supply any reasonable information which may be desired.

UNIVERSITIES AND CORPORATIONS WHICH CONFER DEGREES.

The University of Oxford.
" " " Cambridge.
" " " London.
" " " Edinburgh.
" " " Dublin.
" " " Victoria (Manchester).
" " " Durham (Newcastle-on-Tyne).
" " " Glasgow.
" " " Aberdeen.
" " " St. Andrews.
The Royal College of Physicians (London).
" " " Surgeons (London).
" " " Physicians (Edin.).
" " " Surgeons (Edin.).
Kings and Queens College of Physicians (Dublin).
The Faculty of Physicians and Surgeons (Glasgow).
The Apothecarys' Society (London).

CORPORATIONS AND HOSPITALS WHERE MEDICAL EDUCATION IS GIVEN.

The University of Oxford.
" " " Cambridge.
" " " Edinburgh.
" " " Dublin.
" " " Victoria (Manchester).
" " " Durham (Newcastle-on-Tyne).
" " " Glasgow.
" " " Aberdeen.
St. Bartholomew's Hospital (London).
Guy's " "
St. Thomas's " "
Charing Cross " "
The London " "
Middlesex " "
St. George's " "
St. Mary's " "
King's College " "
University College " "
Westminster " "
The London School of Medicine for Women (30, Handel Street).
Queen's College Hospital (Birmingham).
Leeds (Yorkshire) College.
Liverpool University College.
Manchester (Victoria University).
Newcastle-on-Tyne (Durham University).
Sheffield School of Medicine.
The Bristol Hospital.
Anderson's College (Glasgow).

At most or all of these universities and medical schools a full curriculum is given in medicine, surgery, and midwifery, and to a certain extent the lectures and classes at all of them are recognised by all the degree-conferring bodies. The class fees vary a good deal at the different institutions, and the opportunities for scientific study and bed-side work vary also. The large number of the schools, their localisation in all parts of Great Britain, and their varying cost, bring a complete medical education within the range of, practically, all classes of Her Majesty's subjects.

### SYPHILIS: ITS TRANSMISSION AND TREATMENT
*(continued)*.

By C. GORDON BRODIE, F.R.C.S., Assistant Surgeon to the North-West London Hospital; Senior Demonstrator of Anatomy, Middlesex Hospital.

*Abstract of a paper read before the Middlesex Hospital Medical Society.*

We next come to the third way in which the disease is transmitted namely, from parent to offspring, or by heredity. Any of those who have worked at all in the baby departments of hospital life will recollect the typical "old man babies," with wizened features, broad and sunken noses, large heads, tending to hydrocephalus, stomatitis, snuffling at the nose, often so bad as to make the parent complain that it cannot breathe when suckling; a coppery stain on the buttocks, and perhaps a roseola eruption on the bodies. Badly nourished, and in many cases suffering from intestinal derangements. Such is the most common type which is seen, but there is another which, if not so often seen, is apt to lead to a fallacious diagnosis. I refer to those children who, though pallid, are plump and well nourished, and do not bear any evidence of the disease in their faces; but oftentimes an examination will show a mucous patch at the anus. It is in this latter class of cases that we are apt to be misled; the former are unmistakeable. The most favourable period for the reproduction of this disease in the child is during the secondary or blood infective stage, *i.e.*, when either parent has active syphilis. When it is transmitted from father to mother shortly before conception, the child will inherit its full share of the disease, if it is not fatal before birth. It is a curious fact that this disease is much more likely to be given to the child when the mother has the disease active, than in the case of the father. An interesting fact which arises with this question of heredity is, where the father has no active syphilis, no outward sign of the disease, and yet his wife and child are infected. How does this occur? The man is known to have had syphilis, but the disease is latent, and has been so for some time. Many would say, it was through the medium of the semen that the disease was carried, but of this there is no satisfactory proof. Although a great many attempts have been made by inoculation experiments to produce the disease through this medium they have all fallen through. And here I should like to suggest what strikes me as a probability, although I speak with caution, as I have never had the opportunity to corroborate this opinion. If we were to look with an endoscope along the course of the urethra belonging to an individual who had the latent disease, and had procreated it, might we not find there in some portion a slight erosion, perhaps, of the passage, or maybe a small mucous patch nestling in the corner of the lacunæ, which, by secreting its own ichorous discharge, may be all that is requisite in producing the infection. I have no case to prove this, but I shall quote the case of a gentleman who had procreated a healthy child without a sign of syphilis, and in whom the disease was latent. When his child was four years old he kissed him, having at that time a small sore on the angle of the mouth, and in this way gave him the disease. I should like to deduct from this that a similar state of things might be present in the urethra of those who had infected the wife and child, although the disease was latent in other respects, and it would also help to show how the inoculation of the semen failed to produce the disease, because the urethra was healthy in those cases from which it was taken. There is yet another question of interest to be solved, viz., Is the mother infected first and the child second, or the child first and the mother second? The greater number of the facts at our disposal go far to show that it is the mother who is primarily affected while the child follows suit. In support of the latter view, namely, that the child was infected first by the semen, and then transmitted the poison to the mother are the following: (1) That the ovum was infected by the semen directly; but against this is the experimental fact that the semen does not appear to carry the poison; (2) That in a certain number of the syphilitic miscarriages the fœtal portion of the placenta is affected with syphilitic degeneration, while the maternal has but little or escapes; but supposing that the placenta is only a new growth, formed for a time to be ultimately discarded, surely it is much more likely to become affected with degeneration than the maternal structures which have had time to develope; and, again, the maternal portion would be bathing the fœtal portion with blood imbued with the poison, and so tend more quickly to produce in it a diseased condition than would occur in a less vascular and fully-developed structure. (3) Another point which has been adduced to show that the mother is infected from the child is that often, although a syphilitic child is born, the mother appears to escape, and has no manifestation of the disease herself. On looking more closely at these cases, the mother's health is found to be bad during the period of utero-gestation, becoming debilitated, anæmic, and having numerous glandular swellings, which yield to anti-syphilitic treatment.

and to nothing else; thus proving the truth of the observation that they "merely do not always betray their disease at the time of their lying-in" (Berkley Hill). Another fact, which is even more strong, is that of Colles' law, which was written in 1837, and has never been overturned to this day. It runs thus: "I have never seen or heard of a single instance in which a syphilitic infant (although its mouth be ulcerated), sucked by its own mother, had ever produced ulceration of her breast, whereas very few instances have occurred in which a syphilitic infant had not infected a strange or hired nurse, who had previously been in good health." These facts appear to me to be conclusive, that the mother is infected first, and the fœtus follows. It is rare for a child to escape syphilis should the mother have been infected shortly before impregnation, and it is almost invariably fatal to the child; but after the third month of utero-gestation the inheritance becomes less severe until about the seventh or eighth month, when the transmission usually ceases. Ricord states that it is unusual after the sixth month, but the seventh month is the safer limit, occasionally occurring in the early part, but never in the later. Fournier has noticed that a syphilitic woman who becomes pregnant is more likely to miscarry than a pregnant woman who becomes syphilitic. As soon as the tertiary stage is reached the tendency to transmit the disease gradually dies down until it ceases, the course of the disease being a progression towards recovery. It has been concluded by Weil that the time over which transmission may occur without any treatment at all is a minimum period of seven years and a maximum period of ten. The effect of a mercurial course being to shorten this period very considerably, and when given in a syphilitic woman who is pregnant, in the early stages of utero-gestation, may succeed in helping them to bear a healthy child, controlling for a time the action of the virus, although it is insufficient to eradicate the maternal disease. Fournier quotes the following case, where a syphilitic woman had seven miscarries, took a course of mercury, and bore number eight a healthy child, the ninth also healthy, drop the course, the tenth was syphilitic, the last under mercury was again fairly healthy. There is yet another class of cases of hereditary disease, in which after some, perhaps, slight manifestations in early infancy, the disease becomes latent, but again springs into activity during the period of puberty, attacking the eyes and bones, &c. There was a point which I omitted in the previous paper when speaking of the mediate forms of transmission, viz., that of circumcision, which has often been credited with the means of spreading the disease. If done, as it is often among the Jewish children, by the Rabbi, with a box of instruments such as I have seen produced, I can easily imagine that it is a possible cause of infection, and for the septic condition and terribly unclean state of both box and instruments, I don't think I have seen an equal, hardly in the dissecting-room. However, there are but very few cases on record in which the disease has been transmitted, and the epidemics which have been put down to this cause have turned out nothing more than would be caused by dirt and insufficient attention afterwards. There may be one or two isolated cases on record, but I can find nothing more.

(*To be continued.*)

### APPOINTMENTS.

At St. Thomas's Hospital, C. R. Box, B.Sc. Lond., L.R.C.P., M.R.C.S., and T. H. Kellock, M.A., M.B., B.C., Cantab., F.R.C.S., L.R.C.P., have been appointed resident house physicians; G. R. F. Stilwell, L.R.C.P., M.R.C.S., L.S.A., and D. F. Shearer, B.A., B.M., B.Ch. Oxon., non-resident house physicians; L. Cobbett, F.R.C.S., L.R.C.P., T. H. Haydon, B.A., M.B., B.C. Cantab., L.R.C.P., M.R.C.S., J. R. Harper, L.R.C.P., M.R.C.S., C. Wyman, M.A., M.B., B.C. Cantab., L.R.C.P., M.R.C.S., have been appointed house surgeons; W. F. E. Milton, L.R.C.P., M.R.C.S., and T. A. M. Forde, L.R.C.P., M.R.C.S., assistant house surgeons; W. G. G. Stokes, B.A., M.B., B.C. Cantab., L.R.C.P., M.R.C.S., senior, and A. Banks, L.R.C.P., M.R.C.S., junior obstetric house physicians; P. J. Atkey, L.R.C.P., M.R.C.S., clinical assistant in the throat department; W. F. Umney, L.R.C.P., M.R.C.S., clinical assistant in the eye department; and W. P. Purvis, L.R.C.P., M.R.C.S., clinical assistant in the skin department. At the Birmingham City Asylum, Albert J. Chambers, M.B., B.Ch., M.R.C.S., L.R.C.P., has been appointed clinical assistant, and Mitchel Grabham M.A., M.B., B.Sc., assistant medical officer. At the Woodhouse Schools of the Leeds Union, Samson G. Moore, M.B.B.S., has been appointed assistant medical officer.

### VACANCIES.

The following vacancies are announced this week, the amount of salary in each case being given after the name of the institution:

### Resident Medical Officers.

Bethlem Hospital, S.E., Two Resident Clinical Assistants—Board, &c.

City of London Hospital for Diseases of the Chest, Victoria Park, E., House Physician—Board, &c.

Halifax Infirmary and Dispensary, Assistant House Surgeon—Salary £50, with board, &c.

Islington Dispensary, 303, Upper Street, Islington, Resident Medical Officer—Salary £200, with rooms and cab fares.

Kent County Asylum, Barming Heath, Maidstone, Senior Assistant Medical Officer—Salary £250, increasing to £300.

Royal National Hospital for Consumption, Ventnor, Isle of Wight, Resident Medical Officer—Salary £100, with board, &c.

Royal United Hospital, Bath, House Surgeon—Salary £60, and board, &c.

Scarborough Hospital and Dispensary, House Surgeon—Salary £80, and board, &c.

Stourbridge Dispensary, House Surgeon and Secretary—Salary £130.

West London Hospital, House Physician and House Surgeon—Board, &c.

### Non-Resident Medical Officers.

City Dispensary, Watling Street, E.C., Surgeon.
Dengal Hospital of London, Anæsthetist and Assistant Anæsthetist.
Great Northern Central Hospital, Anæsthetist.
Queen's College, Birmingham, Lecturer on Operative Surgery.
Queen's Colleges, Ireland, Professor of Chemistry.
University of Aberdeen, Six Examiners in Medicine—Salary £30.

### PASS LISTS.

### Royal Colleges of Physicians and Surgeons.

The following gentlemen passed the first examination of the Board in elementary anatomy and elementary physiology at the quarterly meeting of the examiners: Messrs. F. M. Aldred, student of University College, Liverpool; G. Aldridge, London Hospital; J. A. Angus, Middlesex Hospital; H. W. Ashworth, Owens College, Manchester; C. Basan, Middlesex Hospital; A. C. Bean, University College; E. J. Beards, Queen's College, Birmingham; J. R. Benson, King's College; H. Bentley, Owens College, Manchester; E. H. Birchall, University College, Liverpool; J. Blackwood, University College; V. J. Blake, University College; C. S. Bond, Cambridge University; W. E. Bracey, Queen's College, Birmingham; T. W. N. Barlow, University College, Liverpool; J. E. G. Caverley, St. Bartholomew's Hospital; F. J. H. Cann, Guy's Hospital; R. W. S. Christmas, Charing-cross Hospital; H. J. Clague, Sheffield Medical School; E A. Clark, Bristol Medical School; R. F. Clark, Guy's Hospital; E. T. Coady, Dublin; W. L. Cockcroft, Owens College, Manchester; J. H. Cook, University College; T. G. D. Cooper, Cork; P. S. Crookes, St. Thomas's Hospital; E. E. Crowther, Yorkshire College, Leeds; W. Curtis, Owens College, Manchester; Norman Devereux, Cambridge University; H. W. Eldred, St. Thomas's Hospital; E. E. Ellery, Middlesex Hospital; J. L. Elliott, Yorkshire College, Leeds; E. Fielden, Cambridge University and Owens College, Manchester; A. C. Fry, Guy's Hospital; P. V. Fry, Yorkshire College, Leeds; C. W. Gibson, Guy's Hospital; H. T. Gillett, Oxford University; J. A. K. Griffiths, University College; S. G. Harding, Middlesex Hospital; R. E. Harrison, Charing-cross Hospital; W. W. Hoare, Edinburgh University; A. Hodge, Owens College, Manchester; T. E. Holman, Guy's Hospital; J. G. Holmes, Sheffield School of Medicine; T. Hood, St. Bartholomew's Hospital; J. Hora, Guy's Hospital; D. Horwitch, Queen's College, Birmingham; H. Hughes, University College, Liverpool; A. L. Jackson, Cambridge University; K. M. Johnson, St. George's Hospital; A. T. Jones, University College, Liverpool; F. J. Keats, Middlesex Hospital; P. Kitchin, Yorkshire College, Leeds; R. G. Knox, King's College Hospital; E. Leach, Owens College, Manchester; G. A. Leon, London Hospital; E. A. Lermitte, Durham University and St. Bartholomew's Hospital; L. Lloyd, University College; P. G. Lodge, Yorkshire College, Leeds; F. Loud, Guy's Hospital; P. Macaulay, Yorkshire College, Leeds; T. MacDowell, Belfast; R. A. MacLeod, Westminster Hospital; J. W. Maskell, University College, Liverpool; E. C. Montgomery, Charing Cross Hospital; Eugene Moore, Guy's Hospital; Frederick Moreton, Melbourne University and Westminster Hospital; Robert Harry Mornement, Middlesex Hospital; George Yule Myrtle, Yorkshire College, Leeds; H. M. Nicholls, Guy's Hospital; W. T. Nicklin, St. Thomas's Hospital; L. W. Oliver, St. Mary's Hospital; J. C. Padwick, St. Bartholomew's Hospital; A. F. Palmer, Middlesex Hospital; E. M. B. Payne, London Hospital; F. A. Phillips, St. George's Hospital; G. W. Penbury, St. Bartholomew's Hospital; M. B. Pinchard, Middlesex Hospital; H. L. Porteous, Middlesex Hospital; E. Pratt, St. Bartholomew's Hospital; J. P. Prell, London Hospital; C. F. Pritchard, King's College; L. G. Reynolds, Guy's Hospital; E. Lloyd Roberts, University College, Liverpool; A. E. Rutherford, Westminster Hospital; T. H. Sanderson, St. Bartholomew's Hospital; H. M. Sayer, St. George's Hospital; A. R. H. Skey, St. Bartholomew's Hospital; G. U. Smith, King's College; R. B. Stamford, Guy's Hospital; J. R. Steinhaeuser, Guy's Hospital; H. G. W. Stewart, University College; R. L. Storrar, St. Thomas's Hospital; A. B. R. Sworn, University College; A. D. Thomson, St. George's Hospital; G. S. Thompson, Guy's Hospital; R. S. Thorpe, Owens College, Manchester; W. H. Tomlinson, Owens College, Manchester; M. E. Tresider, Guy's Hospital; C. E. Walker, St. George's Hospital; F. E. Walton, S. Thomas's Hospital; H. J. Watts, Owens College, Manchester; T. H. Wells, Middlesex Hospital; M. Wheeler, St. Thomas's Hospital; A. Whitfield, Owen's College, Manchester; J. M. Wilson, Middlesex Hospital; R. A. Wilson, Durham University; W. Wilson, London Hospital; C. P. Windle, Owens College, Manchester; and H. M. Wise, Guy's Hospital.

The following passed in elementary anatomy only, viz.: Messrs. H. N. Collier, Guy's Hospital; W. F. Chrispin, Yorkshire College, Leeds; S. J. R. de Brunet, St. Mary's Hospital; D. Fletcher, St Bartholomew's Hospital; H. Fulton, Guy's Hospital; J. Graham, Dublin; F. G. Haywood, Queen's College, Birmingham; H. C. Holden, Guy's Hospital; J. J. Ireland, University College, Liverpool; F. P. Monckton, Bristol Medical School; H. K. Palmer, St. Bartholomew's Hospital; W. G. Parkinson, Yorkshire College, Leeds; R. S. Ringland, University College, Liverpool; and J. W. Treherne, Guy's Hospital.

The following passed in elementary physiology only, viz.: Messrs. J. M. Adam, University College, Liverpool; H. G. Aspland, London Hospital; E. C. W. Beasley, St. Mary's Hospital; F. V. Bice, St. Bartholomew's Hospital; F. A. R. Brooke, London Hospital; J. Craig, Edinburgh University; T. H. Evans, University College, Liverpool; C. F. Gordon, St. Bartholomew's Hospital; E. G. Grove, St. Mary's Hospital; C. R. L. Harvey, University College; M. B. Johnson, Cambridge University; F. G. P. Laslett, St. Thomas's Hospital; M. M. Lowsley, Charing Cross Hospital; G. M. MacDonald, Cambridge University; W. A. E. Parrott, St. Thomas's Hospital; S. Potter, Edinburgh University; W. St. A. St. John, St. Mary's Hospital; T. B. Sellors, Middlesex Hospital; C. W. Smith, University College; and W. H. Stott, Owens College, Manchester.

